# Enhancing multilevel tea leaf recognition based on improved YOLOv8n

**DOI:** 10.3389/fpls.2025.1540670

**Published:** 2025-03-28

**Authors:** Xinchen Tang, Li Tang, Junmin Li, Xiaofei Guo

**Affiliations:** ^1^ School of Mechanical Engineering, Xihua University, Chengdu, China; ^2^ School of Automobile and Transportation, Xihua University, Chengdu, China

**Keywords:** tea recognition, efficient feature fusion, loss function, smart agriculture, YOLOv8 improvement

## Abstract

In the tea industry, automated tea picking plays a vital role in improving efficiency and ensuring quality. Tea leaf recognition significantly impacts the precision and success of automated operations. In recent years, deep learning has achieved notable advancements in tea detection, yet research on multilevel composite features remains insufficient. To meet the diverse demands of automated tea picking, this study aims to enhance the recognition of different tea leaf categories. A novel method for generating overlapping-labeled tea category datasets is proposed. Additionally, the Tea-You Only Look Once v8n (T-YOLOv8n) model is introduced for multilevel composite tea leaf detection. By incorporating the Convolutional Block Attention Module (CBAM) and the Bidirectional Feature Pyramid Network (BiFPN) for multi-scale feature fusion, the improved T-YOLOv8n model demonstrates superior performance in detecting small and overlapping targets. Moreover, integrating the CIOU and Focal Loss functions further optimizes the accuracy and stability of bounding box predictions. Experimental results highlight that the proposed T-YOLOv8n surpasses YOLOv8, YOLOv5, and YOLOv9 in mAP50, achieving a notable precision increase from 70.5% to 74.4% and recall from 73.3% to 75.4%. Additionally, computational costs are reduced by up to 19.3%, confirming its robustness and suitability for complex tea garden environment. The proposed model demonstrates improved detection accuracy while maintaining computationally efficient operations, facilitating practical deployment in resource-constrained edge computing environments. By integrating advanced feature fusion and data augmentation techniques, the model demonstrates enhanced adaptability to diverse lighting conditions and background variations, improving its robustness in practical scenarios. Moreover, this study contributes to the development of smart agricultural technologies, including intelligent tea leaf classification, automated picking, and real-time tea garden monitoring, providing new opportunities to enhance the efficiency and sustainability of tea production.

## 1 Introduction

The rapid development of modern agriculture is driving advancements in automation and intelligence, where smart equipment significantly enhances production efficiency. As the world’s largest producer and consumer of tea, China holds a significant economic position in the global tea industry. In 2023, tea production in China has reached 3.55 million tons, yielding a market value of 351.18 billion yuan, which is projected to grow to 530.96 billion yuan by 2028. Additionally, the aggregated agricultural output associated with tea has been valued at 329.7 billion yuan (Zhang W. et al., 2024; Liu et al., 2018; Zhao Y. et al., 2022; Huang, 2018).

In recent years, China has established a modern tea industry system encompassing tea culture, scientific innovation, and ecological sustainability. The emerging of agricultural technology has positioned automated tea picking as a key focus for industry innovation. By employing advanced machine vision and sensing technologies, automated tea picking equipment enables precise identification of tea leaves and selective harvesting based on preset standards. This facilitates operational efficiency while preserving the quality of harvested leaves. Compared to traditional manual picking, automated systems offer continuous operation, substantially lower labor costs, and minimize potential damage to tea leaves, making them particularly valuable for producing high-quality tea.

Although current tea leaf recognition technologies achieve satisfactory performance in controlled scenarios, significant challenges still remain when applying these technologies to the intricate environments of tea plantations. For example, variations in lighting, dense foliage, and morphological similarities between tea buds and leaves frequently lead to false detections or missed targets in detection systems (Jie et al., 2023; Li et al., 2024a; Wu et al., 2024). Furthermore, traditional image processing techniques struggle to handle overlapping tea buds and leaves, and their limitations in categorizing tea grades renders them inadequate for the precision required in practical picking processes.

To overcome these challenges, developing robust detection algorithms capable of handling complex scenes is crucial. Modern target detection models can be broadly categorized into two types: single-stage and two-stage approaches (Kang, 2022). Single-stage method, such as You Only Look Once (YOLO) (Sun et al., 2022) and Single Shot MultiBox Detector (SSD) (Lenatti et al., 2023), perform detection in a single forward pass, resulting in high computational efficiency. In contrast, two-stage algorithms, exemplified by Faster R-CNN (Wang et al., 2023), initially generate region proposals and subsequently classify and refine bounding boxes. While two-stage methods excel in detecting small targets and operating in complex environments, they often suffer from reduced computational efficiency due to their two-stage processing pipeline.

In recent years, the YOLO series models have been widely adopted in target detection, promoting the development of smart agriculture (Liu and Wang, 2020; Yu et al., 2024; Lawal et al., 2024). To overcome the challenges of leaf shading and limited computational resources in weed detection, Ren et al. (2024) proposed an enhanced YOLOv8-based algorithm that incorporates large kernel convolution and multi-scale expansion convolution into an expansion feature integration block, significantly improving feature extraction capabilities. For intelligent wheat monitoring, Zhao F. et al. (2022) introduced a YOLOv4-based method that integrates spatial pyramid pooling (SPP) blocks, addressing data acquisition difficulties and enhancing detection accuracy. In crop disease detection, Zhao et al. (2023) developed an improved YOLOv5s model by optimizing the feature fusion structure and introducing a CAM mechanism, thereby boosting both robustness and precision in detecting small and densely packed targets. Similarly, Peng and Wang (2022) designed an innovative image retrieval system integrated with YOLOv5 to improve small target recognition, enabling automatic disease identification in open environments. In the domain of yield estimation, Zhang M. et al., 2024; Peng and Wang, 2022) presented a depth-aware detection model based on YOLOv8, which employs SPD-Conv and a small target detection head to significantly improve detection performance, providing reliable solutions for high-precision cotton monitoring and yield estimation. These studies highlight the versatility and effectiveness of YOLO-based models in addressing diverse challenges in smart agriculture, ranging from improving disease detection to enabling efficient yield assessment.

Meanwhile, in tea leaf recognition, researchers have proposed various methods to address challenges posed by the complex background of tea buds. Xie and Sun (2023) introduced Tea-YOLOv8s, a model that enhances detection accuracy through data augmentation and the integration of deformable convolution and attention mechanisms, achieving an average accuracy of 88.27% in tea bud detection. To resolve resource constraints on edge devices, Zhang S. et al. (2023) developed ShuffleNetv2-YOLOv5-Lite-E, which applies channel pruning and model lightweight optimization to significantly improve detection speed while maintaining accuracy, achieving a detection rate of 8.6 fps on edge devices. Zhu et al. (2023) proposed an enhanced version of YOLOv5-Lite-E tailored for unstructured tea gardens, integrating ECANet and BiFPN modules to boost detection precision. Furthermore, by incorporating the DBSCAN algorithm and principal component analysis, their method enables 3D localization of tea buds, achieving a detection accuracy of 94.4% with an average localization error of 3–7 mm, thus meetings the requirements of tea-picking robots. However, existing studies primarily focus on single-type or graded recognition of tea leaves (Xia et al., 2024). In automated tea-picking processes, current methods often suffer from low efficiency and missed picks. In order to meet diverse picking requirements, it is crucial to develop multi-level composite recognition systems capable of accurately identifying tea leaves, classifying them into detailed grades, and flexibly selecting grades for picking based on demand.

In summary, to tackle the challenges of accurately recognizing tea grades and enabling the flexible selection of picking grades, this paper proposes an improved YOLOv8-based target detection algorithm. It contributes the literature in threefold: (1) During the dataset construction, overlapping labeling of “bud”, “sprout” and “bud leave 2” at the top of the tea plant is employed in building the dataset, so as to achieve the recognition of “one bud and two leaves” and other grades simultaneously on a single tea leaf. (2) The CBAM module is added to the Neck section to enhance feature expression and model robustness, while the Concat section is replaced with the BiFPN module to enable efficient multi-scale feature fusion, thereby improving detection performance for small and overlapping targets. (3) The loss function is modified to a combination of CIOU and Focal Loss, enhancing the model’s ability to handle complex scenes and class imbalances, significantly improving both detection accuracy and robustness.

The remainder of the paper is structured as follows. Section 2 describes the process of dataset collection and the subsequent preprocessing procedures, along with the application of data enhancement techniques. Section 3 presents the specific improvements applied to the YOLO model. Section 4 presents the experimental results and analyzes the results through comparative and ablation studies. Section 5 summarizes the conclusions of this study.

## 2 Tea data set collection and processing

### 2.1 Tea dataset production

The tea dataset utilized in this study was collected from the Daoyuanxianweng Organic Tea Base in Chengdu City, Sichuan Province, China. A total of 3,102 images were captured, covering a wide range of periods and viewing angles. Although all images were taken during the day, the dataset leverages the natural variation in lighting conditions at different times of day—morning, noon, and afternoon—capturing the differences in light intensity caused by changes in the solar angle. Furthermore, to enhance the diversity of the dataset, a multi-angle shooting strategy was employed during image collection, including horizontal, top-down, and tilted perspectives, thus covering various observation directions and field-of-view ranges.

To compensate for the limitations of real-world conditions, online data augmentation techniques were incorporated during model training to simulate environmental changes that may affect the detection task. For instance, adjustments to brightness, contrast, and saturation were made to simulate fluctuations in lighting conditions; Gaussian noise and blur were added to mimic sensor noise and the blurring effects in the imaging process; random rotations, translations, and scaling were applied to simulate the impact of camera movement or angle changes on the targets; and weather effects such as rain, fog, and snow were introduced to simulate potential environmental influences. These measures enhanced the dataset’s visual diversity and improved the model’s generalization ability in complex real-world scenarios.

The dataset was divided into training, validation, and test sets at a ratio of 7:2:1, with the images labeled using the LabelImg tool. The labeling categories included “one bud and one leaf” (bud leaf), “bud” (sprout), and “one bud and two leaves” (bud leaf2), with the annotations were saved in YOLO’s text format. During the labeling process, overlapping annotations were applied to each tea plant, allowing both the buds and leaves of the same plant to be labeled simultaneously. This overlapping annotation method not only enhances the model’s ability to automatically identify the top part of the tea leaves, but also supports more precise harvesting strategies.

In contrast to traditional detection methods, which typically focus on identifying a single category per image or region, the proposed approach introduces an innovative overlapping annotation framework that allows simultaneous detection of multiple categories (bud, one bud and one leaf, one bud and two leaves) within the same tea shoot. This capability enables a more comprehensive characterization of tea leaf structures, offering significant advantages in precision agriculture and tea production. For instance, in growth monitoring, this method provides a detailed distribution of different developmental stages in a single detection pass, facilitating the accurate assessment of tea plant health and growth dynamics. Traditional methods often require repetitive annotation and detection, leading to higher costs and inconsistencies.

In practical applications, this overlapping annotation strategy empowers intelligent harvesting systems to implement customized picking strategies. By leveraging real-time multi-category information, the system can prioritize specific tea parts based on production goals: for instance, focusing on buds for premium tea production or selecting “one bud and one leaf” for mass-market tea. Such flexibility, enabled by simultaneous multi-category detection, optimizes the harvesting process, reduces waste, and ensures product quality. Beyond harvesting, this approach can enhance sorting and classification during processing, where different tea parts necessitate distinct handling techniques. Moreover, in market applications, the method supports automated quality grading and traceability by analyzing the proportions of tea leaf categories, improving efficiency and fairness in tea trade.

This overlapping annotation framework addresses a key limitation of traditional detection methods: their inability to manage the complex diversity within a single target. By providing rich, multi-dimensional data in a single detection pass, the proposed method eliminates the need for multiple rounds of processing and post-detection integration, making it more suitable for real-time intelligent systems. This not only enhances the efficiency of tea production but also offers a scalable solution for integrating artificial intelligence into precision agriculture.

We believe that by implementing the T-YOLOv8n system, tea farmers could see several key benefits. First, it could substantially reduce labor costs. Traditional manual grading is not only labor-intensive but also prone to subjective errors, so automating this process would ease the need for manual labor. This, in turn, would lower costs. Second, the system’s high-precision detection improves the accuracy and consistency of tea grading, helping to ensure more standardized results and minimizing human error. Lastly, with faster and more accurate grading, tea farmers would be able to harvest and process tea more efficiently, ultimately boosting overall production.

However, in the actual deployment of the system, there may be challenges such as initial equipment and deployment costs, as well as the tea farmers’ acceptance of new technologies. These factors require further economic analysis and research in practical applications to evaluate the cost-effectiveness of the system.

### 2.2 Data enhancement methods

In target detection tasks, data augmentation plays a crucial role in enhancing the generalization ability of the model. YOLOv8 supports several data augmentation techniques, which significantly boost model performance in complex scenarios. These methods can be broadly categorized as offline data augmentation, Mosaic augmentation, and online data augmentation.

#### 2.2.1 Offline data enhancement

**Figure 1:**
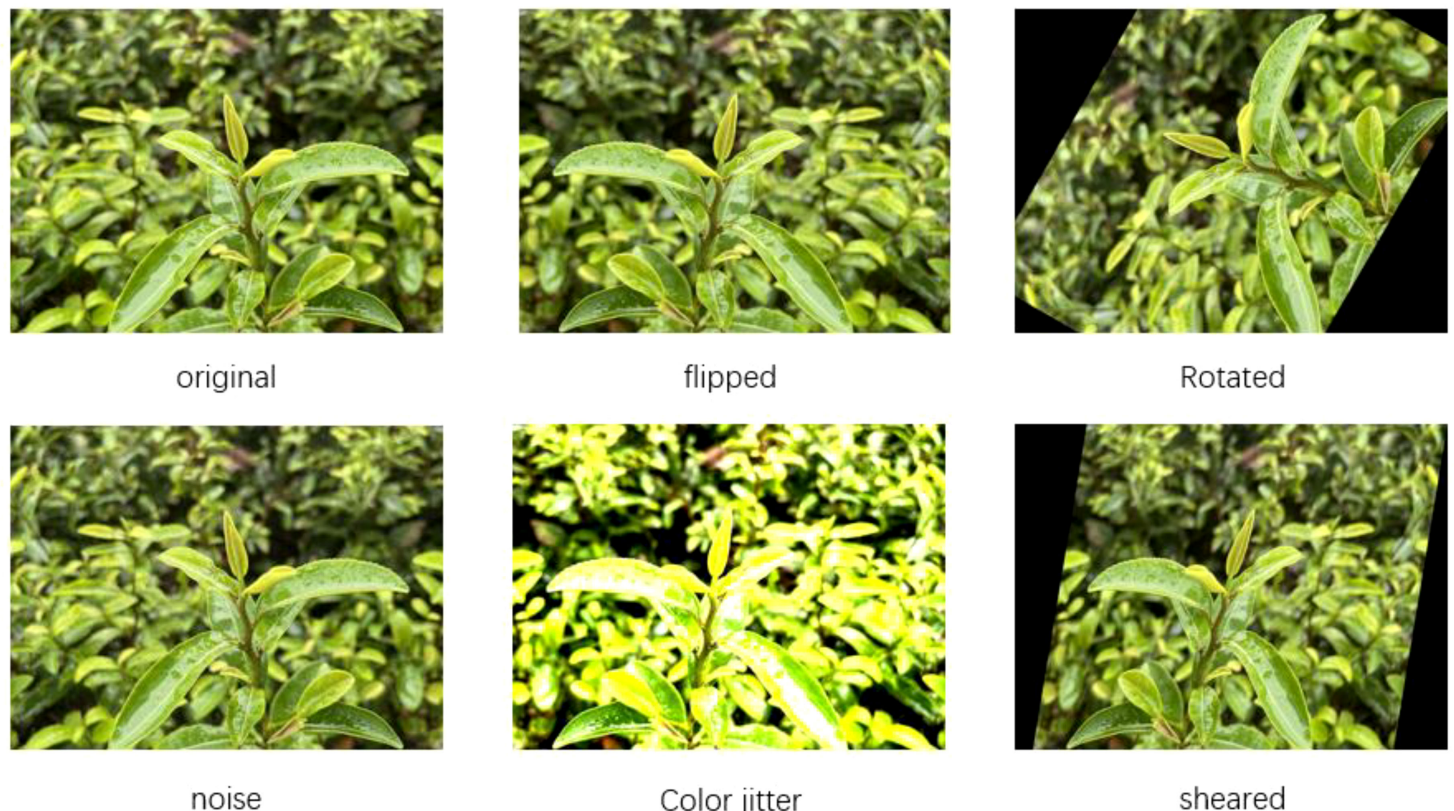
Offline data enhancement schematic.

Offline data augmentation involves applying transformations such as flipping, rotation, scaling, and adding noise to images prior to training. This increases the dataset size and sample diversity, thereby improving the model’s robustness and reducing overfitting. **Figure 1** shows the schematic diagram of offline data enhancement.

#### 2.2.2 Mosaic enhancement

Mosaic enhancement generates diverse training samples by randomly cropping and stitching together four images, followed by scaling and further cropping. This increases background complexity and target variation without significantly enlarging the dataset size. This method enhances the model’s ability to detect small targets and recognize complex backgrounds, which is particularly beneficial for tea leaf recognition tasks.

#### 2.2.3 Online data enhancement

Online data enhancement generates augmented images in real-time during each training cycle. Unlike offline enhancement, its inherent randomness ensures that the model is exposed to a variety of modified data, helping to prevent overfitting. In tea leaf detection tasks, online data enhancement significantly improves the model’s ability to detect small and overlapping targets. Furthermore, dynamic adjustment of the enhancement strategy ensures better adaptability to the diverse shapes of tea leaves and varying background environments, making it particularly suitable for small target detection in complex backgrounds. This paper utilizes the YOLOv8 framework combined with the Albumentations library to implement the online enhancement strategy, which offers a rich set of efficient image augmentation operations. These operations not only improve the model’s generalization but also boost detection accuracy.

**Figure 2:**
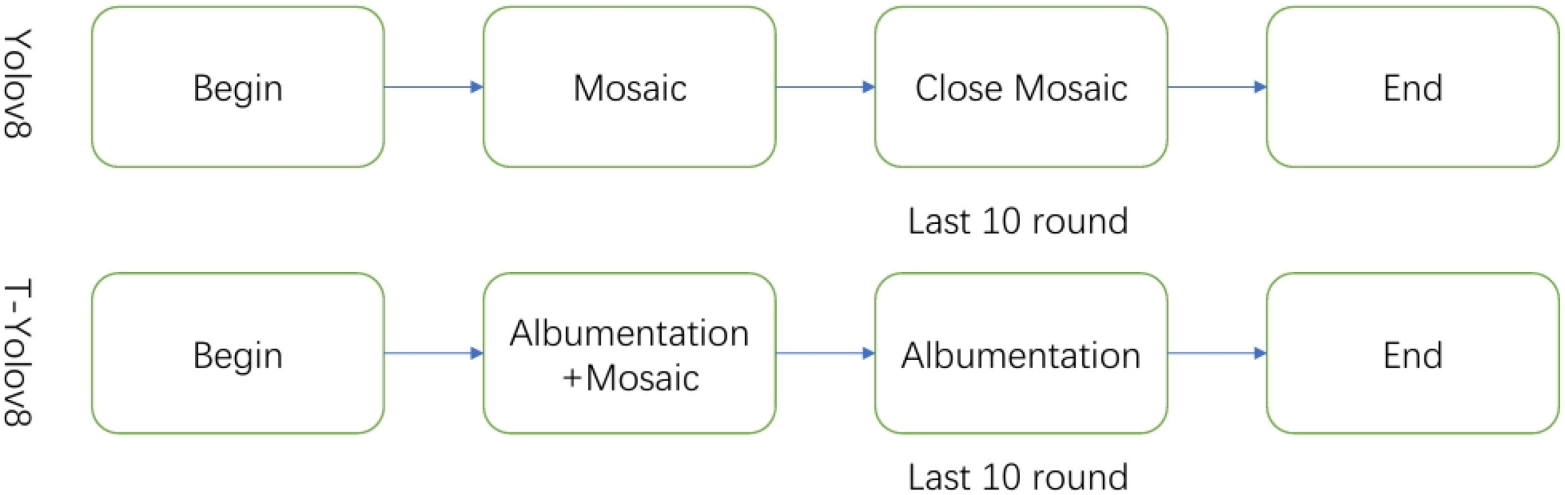
Data enhancement schematic.

As shown in **Figure 2**, offline data enhancement’s static characteristics do not fully meet the dynamic training needs. Additionally, it may consume excessive storage and processing resources as the dataset expands. The Mosaic enhancement technique, while improving sample diversity, may introduce edge deformations that affect bounding box prediction accuracy. Furthermore, incorporating complex backgrounds may cause the model to overly rely on these backgrounds, reducing detection accuracy. To overcome these challenges, this paper combines online data enhancement with Mosaic enhancement strategies. This integrated approach processes the data to enhance the model’s generalization ability and, in turn, improves training accuracy, especially for small target recognition and overlapping bounding boxes in tea leaf recognition.

### 2.3 This article online data enhancement strategies

In this paper, the main categories of online data enhancement strategies are as follows:

Base category: Includes operations such as Blur, MedianBlur, and GaussianBlur. These operations adjust the blur strength through parameters like the kernel size, primarily affecting image clarity and enhancing the model’s noise immunity through moderate blurring.

Spatial: These operations, such as RandomResizedCrop, HorizontalFlip/VerticalFlip, Rotate, and Perspective, modify the spatial structure of the image. For example, the RandomResizedCrop operation allows the model to better adapt to targets at different scales and angles by controlling the size and proportion of the cropped area.

Pixel Class: Includes operations like ImageCompression and ToGray, which simulate varying image qualities or color modes to improve the model’s robustness. Image compression, for instance, enables the model to adapt to varying image qualities.

Weather effects: Operations such as RandomFog, RandomRain, and RandomSnow simulate environmental changes, ensuring the model performs reliably under various weather conditions.

Additional Enhancements: Techniques like Cutout and CoarseDropout simulate missing or occluded data by masking part of the image, enhancing the model’s ability to handle incomplete or occluded features.

The combination of these online enhancement strategies, as detailed in **Table 1**, ensures comprehensive multi-dimensional enhancement for tea leaf detection, thereby improving model generalization and small target recognition:

**Table 1:** Online enhancement strategy sheet.

Strategy	Categories(probability)	Names	Parameters	Probability
Base	Category 1(0.2)	Blur	3,7	0.1
		MedianBlur	3,7	0.1
		GaussianBlur	3,7	0.1
	Category 2(0.4)	RandomBrightnessContrast	0.3,0.3	0.3
		HueSaturationValue	20,30	0.3
		ColorJitter	\	0.3
	Category 3	CLAHE RandomGama	\	0.1
Spatial	Category 1(0.6)	RandomResizedCrop	640,640	0.4
		RandomCrop	640,640	0.4
		Resize	640,640	0.4
	Category 2	HorizontalFlip	\	0.5
		VerticalFlip	\	0.2
		Rotate	\	0.4
		ShiftScaleRotate	0.1,0.2,20	0.4
		Perspective	\	0.3
Pixel	Category 1	ImageCompression	50,100	0.3
		ToGray	\	0.1
Weather	Category 1(0.2)	RandomFog	\	0.1
		RandomRain	\	0.1
		RandomSnow	\	0.1
Additional Enhancement	Category 1	Cutout	8,64,64	0.1
		CoarseDropout	8,64,64	0.1

## 3 Design of improved YOLO algorithm

### 3.1 The yolov8 algorithm and ideas for improvement

YOLOv8 (Yukang et al., 2024; Wang et al., 2024; Solimani et al., 2024), a more advanced version of the YOLO series released in January 2023, introduces several optimizations while preserving its efficient detection capabilities. Firstly, YOLOv8 incorporates a deeper network architecture, allowing the model to extract richer features and thus improving detection accuracy. Additionally, YOLOv8 integrates advanced techniques, including adaptive anchor frame generation, hybrid data augmentation, and enhanced loss functions, which further strengthen the model’s robustness and generalization ability. Moreover, YOLOv8 optimizes inference speed, enabling more efficient target detection with reduced computational resource consumption, making it more suitable for practical applications.

**Figure 3:**
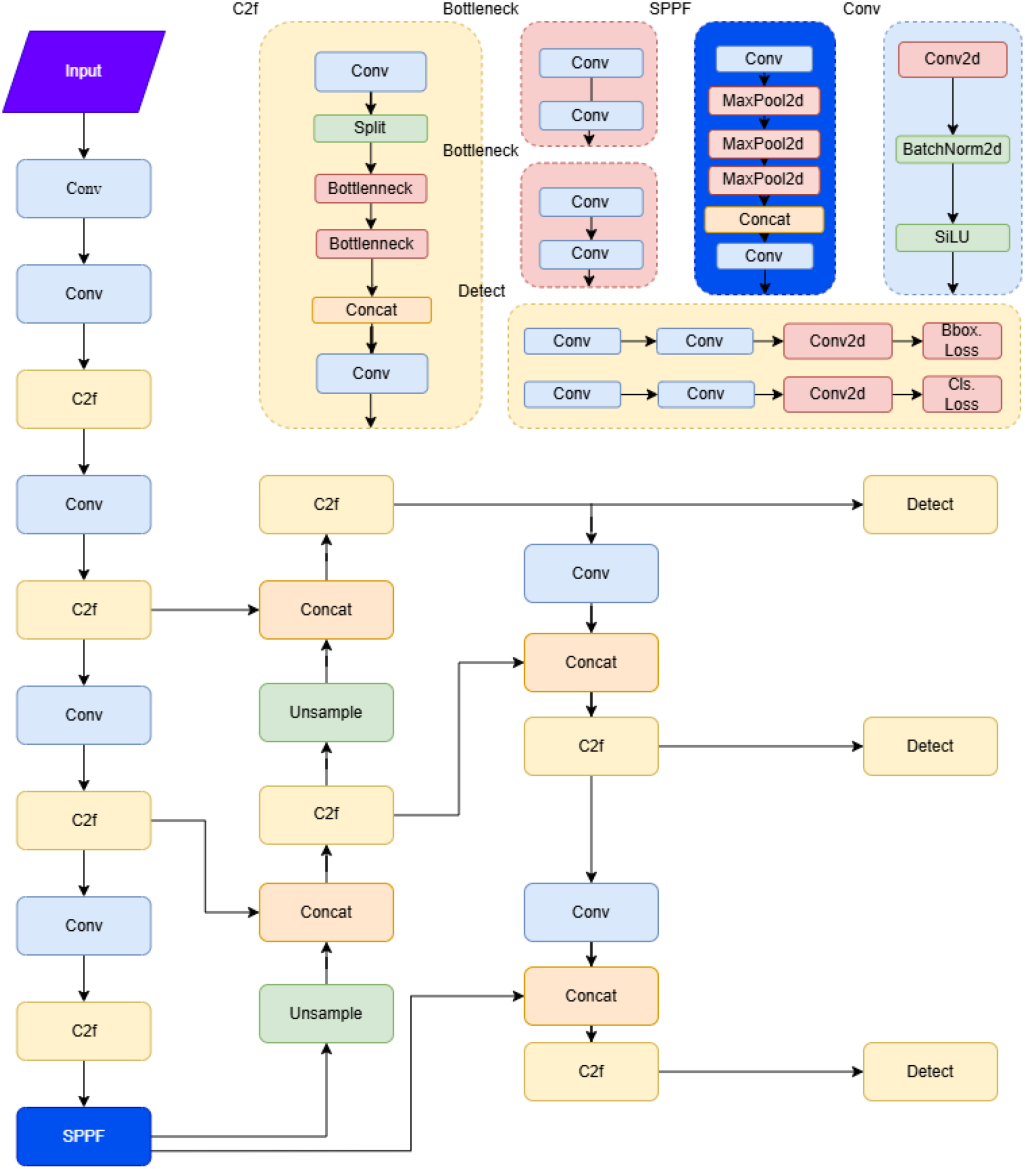
Yolov8 structure diagram.

**Figure 3** shows the underlying framework structure of YOLOv8:

Compared to two-stage detection algorithms like Faster R-CNN, YOLOv8 offers a significant advantage in detection speed, making it more suitable for real-time applications such as autonomous driving, video surveillance, and UAV image analysis. Additionally, YOLOv8 outperforms other single-stage detectors, like SSD, in small target detection and complex background handling.

While YOLO models generally perform well in crop detection, challenges still remain in tea target detection, such as fine-grained feature extraction for tea buds, balancing detection accuracy and speed, and dealing with limited computational resources. YOLOv8 addresses some of these issues with a more lightweight design, making it better suited for edge device deployment, while improving in both detection speed and accuracy.

This paper builds upon YOLOv8 to improve both tea grading and bud detection accuracy and efficiency. The main improvements are as follows: First, the BIFPN-concat2 and BIFPN-concat3 (Zhang H. et al., 2023; Mo and Wei, 2024; Li et al., 2024b) modules replace the standard Concat module in the YOLOv8 backbone, enhancing the model’s performance in processing low-resolution images and detecting small targets. Second, the CBAM (Wang et al., 2024; Liu et al., 2024; Li et al., 2023) module is incorporated to enhance the model’s ability to extract crucial information in complex scenes, thereby improving accuracy in tea grading detection. Finally, the loss function is optimized by replacing the base IoU with CIOU+FOCAL (Gao et al., 2021; Xue et al., 2022; Chen and Qin, 2022; Gu et al., 2024), which enhances bounding box regression and accelerate model convergence.

**Figure 4:**
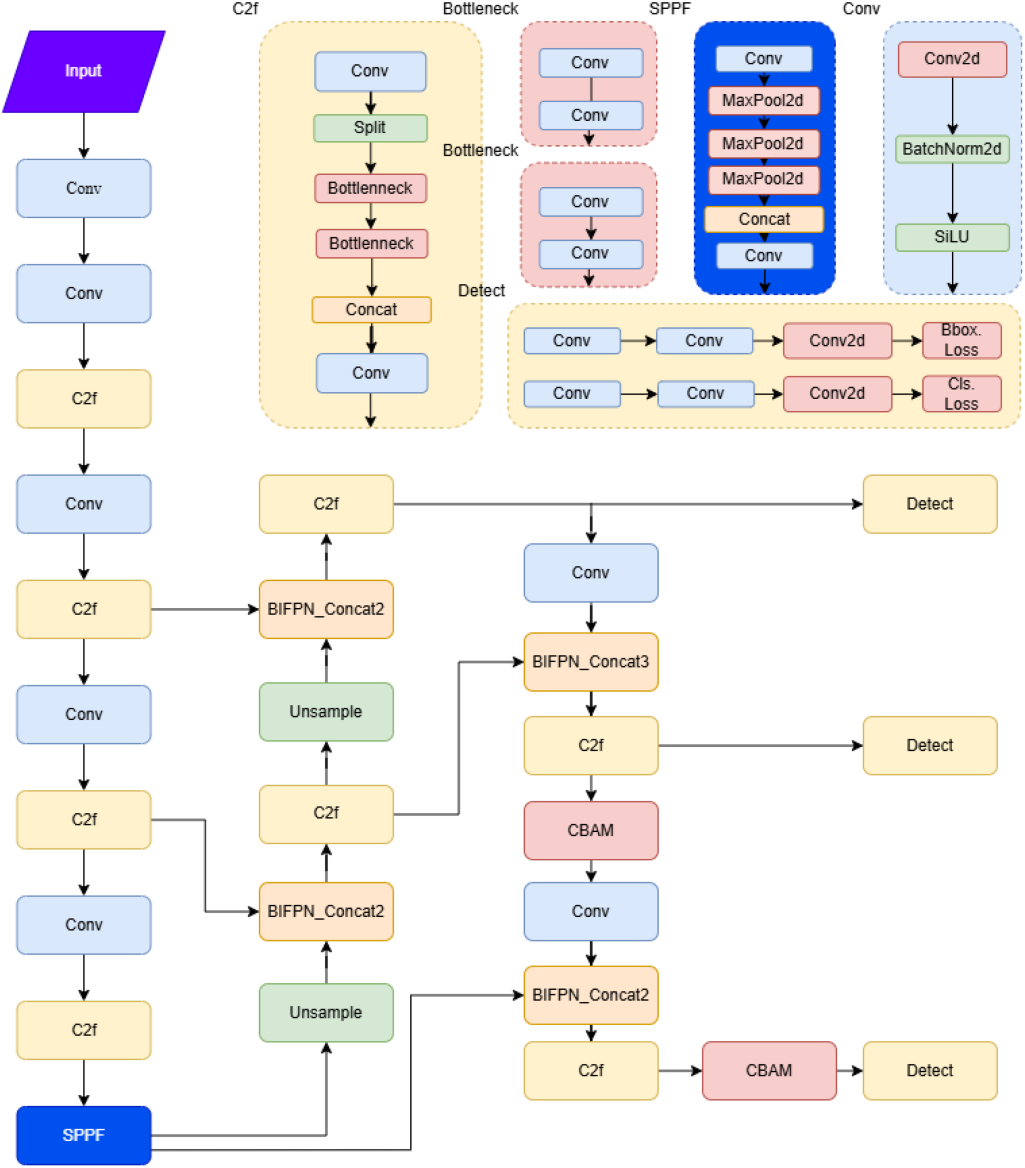
Improved structure diagram.

As shown in **Figure 4**, the CBAM and BIFPN modules are introduced into the neck part of YOLOv8 to enhance the model’s capabilities. The CBAM is placed after the last two C2f layers of the neck, and the initial Concat is replaced with BIFPN-Concat2 and BIFPN-Concat3. The improved network structure is illustrated in the figure, with the red box highlighting the added CBAM module, while the Concat is replaced by BIFPN-Concat2 and BIFPN-Concat3.

CBAM enhances the model’s ability to recognize small targets and objects in complex backgrounds by focusing on more relevant feature regions and channels. This is achieved this through channel and spatial attention mechanisms, which guide the network’s attention towards the most informative parts of the input. In contrast, BiFPN replaces the Concat operation to enable more efficient multi-scale feature fusion. By adaptively adjusting the contribution of feature maps at different resolutions through learnable weights, BiFPN further improves the model’s ability to detect small targets and handle complex environments.

### 3.2 Adding the BIFPN module

BiFPN (Bidirectional Feature Pyramid Network) significantly improves the efficiency of multi-scale feature fusion by enhancing the traditional FPN (Feature Pyramid Network). Its key innovations are as follows:

1) Bidirectional feature fusion: Unlike traditional FPNs, which fuse feature maps from high to low resolution in a top-down manner, BiFPN introduces bottom-up paths for feature fusion. This bi-directional mechanism facilitates more comprehensive multi-scale feature sharing. The higher-level features carry richer information, while the lower-level features retain stronger semantic understanding, enhancing both feature maps. 2) Simplified topology: BiFPN reduces unnecessary edge connections found in FPN, retaining only essential fusion paths. This simplification optimizes, optimizing information transfer and reduces computational costs. 3) Learnable fusion weights: By incorporating learnable weights in the fusion process, BiFPN allows the network to adaptively adjust the contribution of feature maps from different scales based on the specific task. This dynamic adjustment ensures the more effective utilization of multi-scale features during fusion. 4) Efficient multi-scale feature fusion: BiFPN performs multiple up-sampling and down-sampling operations, merging features from various resolutions using weighted fusion. This ensures that the final feature map captures essential information across all scales, improving small target detection and accommodating variations in target sizes.

Due to these improvements, BiFPN enhances the ability of target detection networks’ to detect small and multi-scale targets with minimal computational cost. This structure, which is a core component of the EfficientDet model, has been widely adopted in various detection tasks.

**Figure 5:**
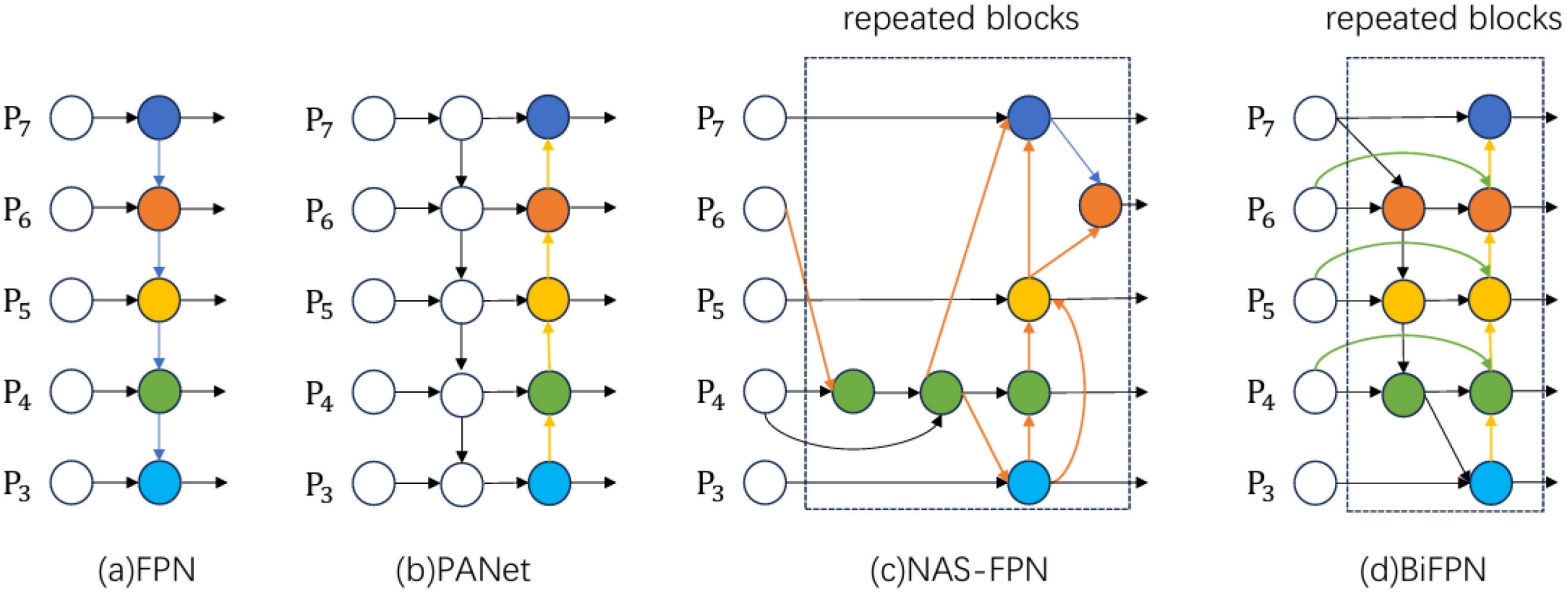
Feature network structure diagram-**(a)** FPN introduces top-down paths to fuse multi-scale features from level 3 to level 7 (P3 - P7); **(b)** PANet adds a bottom-up path to the FPN structure; **(c)** NAS-FPN uses neural architecture search to optimize feature network topology, applying the same blocks repeatedly; **(d)** BiFPN improves on the previous designs with bi-directional cross-scale connectivity and weighted feature fusion, offering better accuracy and efficiency trade-offs.

The comparative diagram of BIFPN structure is shown in **Figure 5**.

### 3.3 Add lightweight CBAM module

CBAM (Convolutional Block Attention Module) is designed to mitigate the limitations of traditional convolutional neural networks when processing information across different scales, shapes, and orientations. To achieve this, CBAM incorporates two attention mechanisms: channel attention and spatial attention. Channel attention enhances the feature representation of different channels, while spatial attention focuses on extracting important information from different spatial locations within the image.

CBAM comprises two main components: the Channel Attention Module (C-channel) and the Spatial Attention Module (S-channel). These two modules can be separately integrated into various layers of the CNN to improve feature representation.

**Figure 6:**
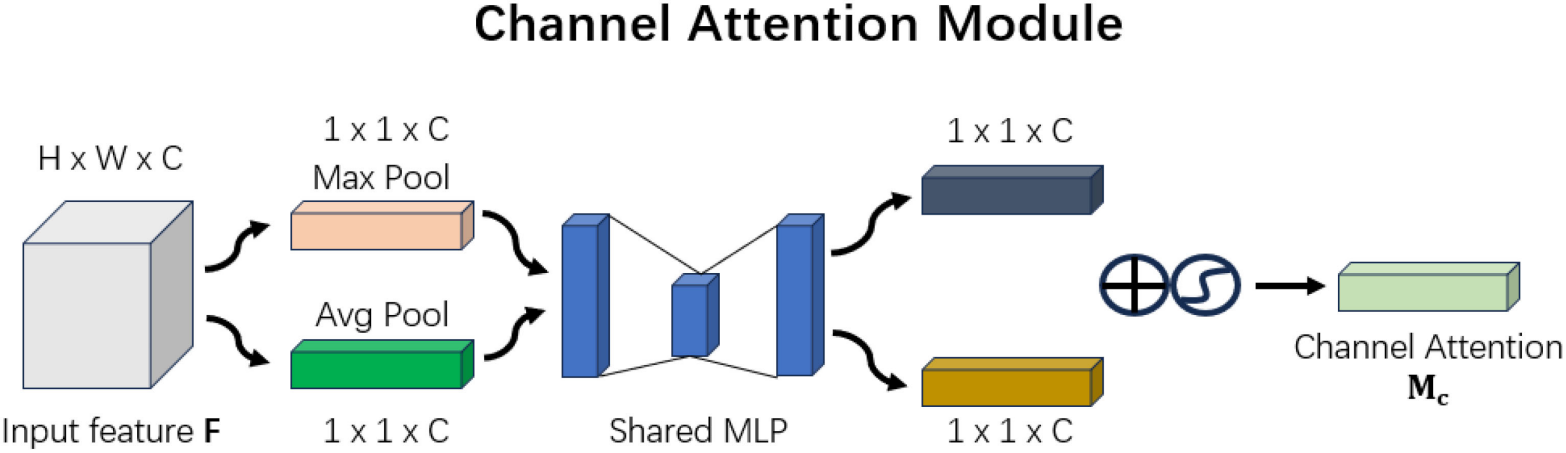
Channel attention module.

The structure of the Channel Attention Module is shown in **Figure 6**.

This diagram, presented in the CBAM paper, illustrates the Channel Attention Module, with feature map dimensions added for clarity. The process begins with global maximum pooling and global average pooling to downsample the input feature map F. The original feature map F, with dimensions H×W×C, is transformed into two 1×1×C feature maps. These two feature maps are then passed through two fully connected layers (MLPs), each producing a 1×1×C output. After obtaining these two 1×1×C feature maps, they are summed and passed through a sigmoid activation function to constrain their values between 0 and 1. The result is the final Channel Attention map, with dimensions of 1×1×C as shown in the figure above. This process can be represented by the following **Equation 1**:


(1) $$M_{c}(F)=\sigma (MLP(AvgPool(F))+MLP(MaxPool(F)))$$

**Figure 7:**
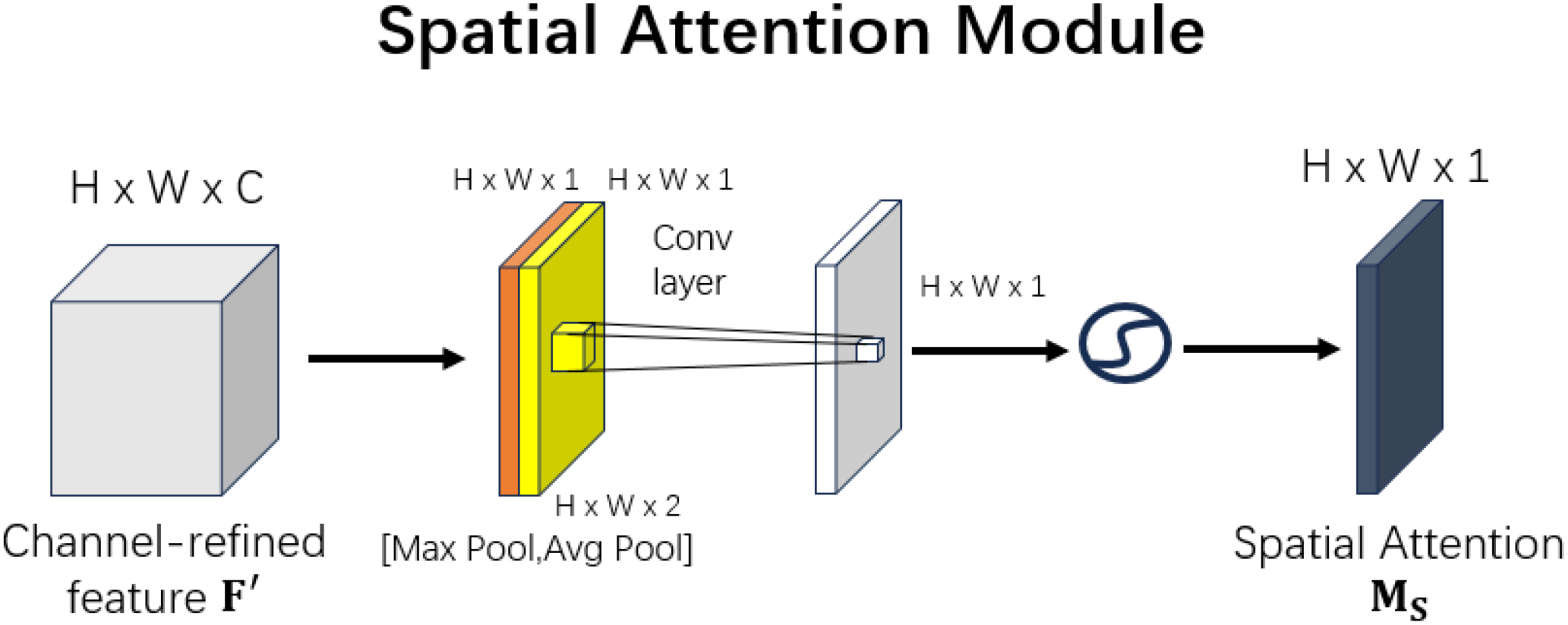
Spatial attention module.

The structure of the Spatial Attention Module is shown in **Figure 7**.

Similarly, the paper presents the structure of the spatial attention module, as shown in the figure. After obtaining the feature map of size H×W×C, the spatial attention module performs two types of pooling operations: global maximum pooling and global average pooling, both applied across the channel dimension. Specifically, global maximum pooling generates a feature map of size H×W×1 (orange), and global average pooling produces a feature map of size H×W×1 (yellow). These two feature maps are then concatenated along the channel dimension, resulting in a combined feature map of size H×W×2.

Subsequently, a convolution operation is applied to reduce the combined feature map from H×W×2 to H×W×1. Finally, a sigmoid activation function is applied to scale the feature map values to the range of 0 to 1, resulting in the final output with a size of H×W×1.

This process is represented by the following **Equation 2**:


(2) $$M_{s}(F)=\sigma (f^{7\times 7}([AvgPool(F);MaxPool(F)]))$$

The above equation indicates that the convolution operation uses a 7×7 convolution kernel.

**Figure 8:**
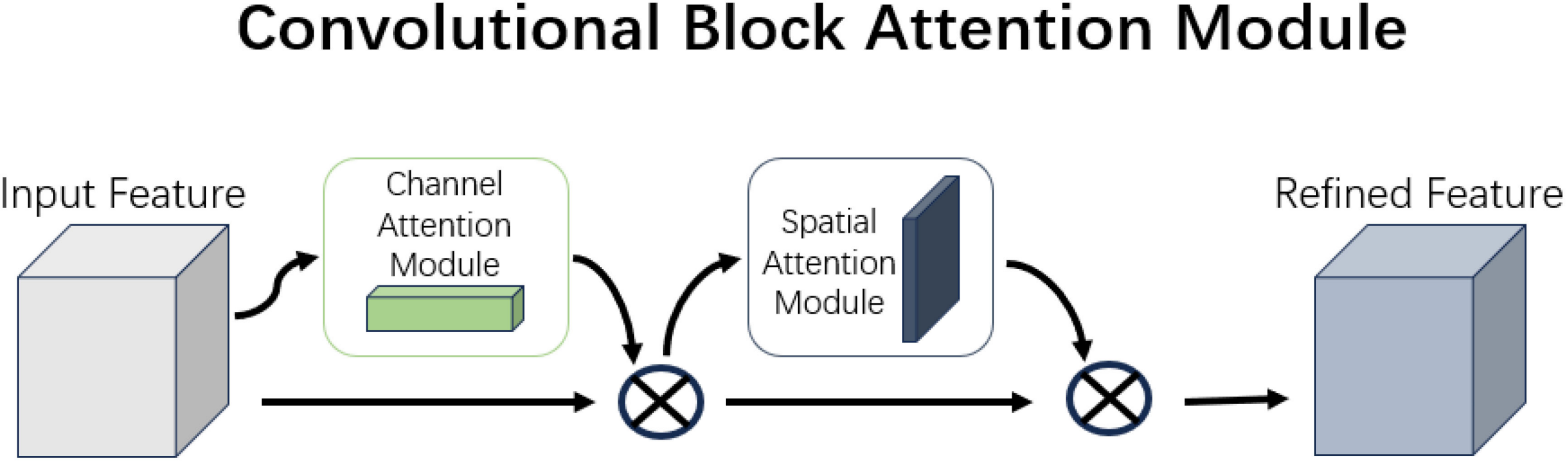
Convolutional block attention module.

The structure of the Convolutional Block Attention Module is shown in **Figure 8**.

CBAM involves element-wise multiplication of the output features from both the Channel and Spatial Attention Modules to generate the final attention-enhanced feature. This enhanced feature is then passed to subsequent layers of the network, helping to suppress noise and irrelevant information while preserving important features. The original experiments integrate channel attention first, followed by spatial attention.

### 3.4 Improvement of the loss function

Default loss function for YOLOv8:

YOLOv8’s default loss function leverages an IOU-based approach to optimize the target detection model by maximizing the overlap between the predicted and true bounding boxes. This loss function consists of three primary components: localization loss, objectness loss, and classification loss. These terms collectively guide the model to refine its predictions in terms of bounding box accuracy, the presence or absence of targets, and correct class identification.

The positional loss in YOLOv8 is based on the Intersection over Union (IOU), a metric that measures the overlap between the predicted and actual bounding boxes. Specifically, IOU is defined as the ratio of the area of overlap between the predicted and true boxes to the area of their union.

The IOU formula is as follows **Equation 3**:


(3) $$IoU=\frac{AreaofOverlap}{AreaofUnion}=\frac{\left|B_{p}\cap B_{gt}\right|}{\left|B_{p}\cup B_{gt}\right|}$$

Where $B_{p}$ denotes the predicted frame and $B_{gt}$ denotes the ground truth frame. The IOU value ranges from 0 to 1, with larger values indicating better overlap between the predicted and true bounding boxes. YOLOv8 optimizes the bounding box prediction by minimizing 1−IOU, driving the predicted box closer to the true box. However, IOU only accounts for the overlapping area of the bounding boxes, ignoring factors such as center distance and shape differences. This limitation can lead to inaccurate bounding box positioning in certain scenarios, where small changes in center or shape may not be reflected in the IOU score.

CIOU (Complete Intersection over Union):

CIOU was introduced to address the limitations of IOU. It improves bounding box prediction accuracy by incorporating additional geometric constraints. The CIOU loss function combines IOU with two important factors: the Euclidean distance between the centroids of the predicted and true bounding boxes, and the aspect ratio difference between them. This makes CIOU a more comprehensive loss function for bounding box regression. The CIOU loss formula is as follows **Equation 4**:


(4) $$L_{\text{CIOU}}=1-\text{IoU}+\frac{p^{2}(b,b_{gt})}{c^{2}}+\alpha v$$

Where IOU represents the intersection-over-union ratio, which quantifies the overlap between the predicted and ground-truth bounding boxes. $p(b,b_{gt})$ refers to the Euclidean distance between their centroids, while c is the diagonal length of the smallest enclosing box that contains both the predicted and ground-truth bounding boxes. v represents the difference in aspect ratios between the two boxes, and α is a balancing factor that adjusts the trade-off between IOU loss and aspect ratio loss.

CIOU enhances bounding box prediction through two key components:

Distance Loss: Represented by $\frac{p^{2}(b,b_{gt})}{c^{2}}$ this term evaluates the ratio of the Euclidean distance between the centroids of the predicted and ground-truth boxes to the diagonal length of their smallest enclosing box. By penalizing predictions far from the true center, it reduces deviations even in cases with high IOU, making CIOU more robust for detecting complex shapes or distant targets.

Aspect ratio loss: Represented by αv , this term measures the difference in aspect ratios between the predicted and ground-truth boxes. By adjusting the weight α , the balance between IOU loss and aspect ratio loss can be fine-tuned, ensuring a closer match between the predicted and actual object dimensions.

**Figure 9:**
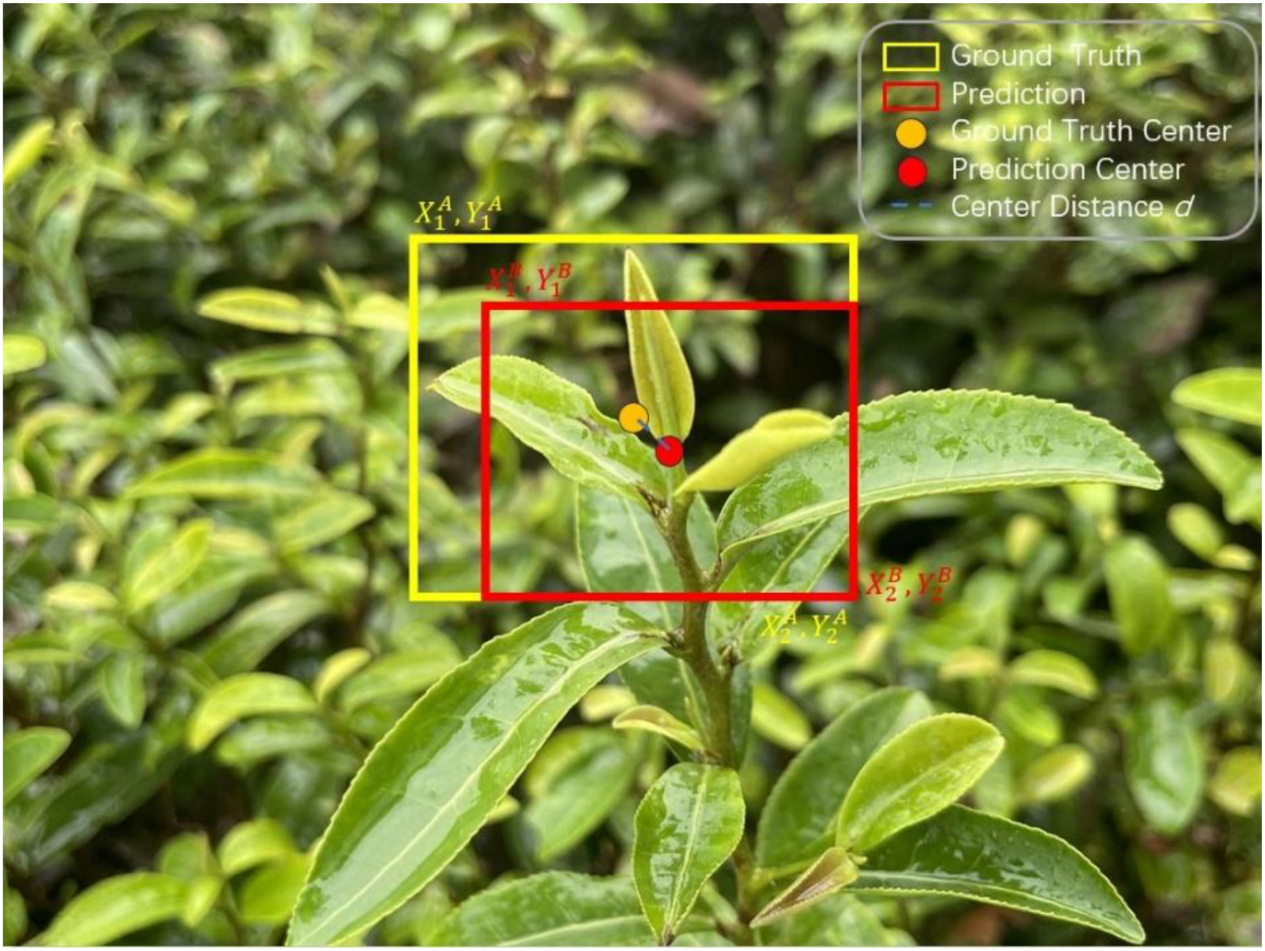
CIOU structure diagram.

The structure of CIOU is shown in **Figure 9**.

In tea leaf detection, the challenges of category imbalance and small target detection are particularly pronounced. For instance, some tea varieties dominate the dataset, while finer targets, such as buds and leaves, constitute only a small fraction of the image. These issues exacerbate detection difficulty, as traditional cross-entropy loss struggles to address category imbalance and fails to emphasize hard-to-classify samples.

To tackle these challenges, Focal Loss is introduced alongside CIOU. Focal Loss down-weights easy samples, directing the model’s focus towards difficult ones. This approach significantly improves detection accuracy for small targets and minority classes. In the context of tea leaf recognition, Focal Loss enhances the model’s robustness across various planting environments, picking methods, and shooting conditions.

In the next section, the principles of Focal Loss will be discussed.

Focal Loss enhances the standard cross-entropy loss by introducing a moderating factor ${\left(1-p_{t}\right)}^{\gamma }$ to adjust the loss contribution of easily categorized samples. Here, $p_{t}$ denotes the added moderating factor formula, designed to reduce the influence of well-classified samples on the loss. $p_{t}$ represents the prediction probability for the correct category, serving as a key input to the moderating factor. γ is a focal parameter, controlling the extent to which the loss contribution from easily categorized samples is down-weighted. By appropriately setting, γ Focal Loss enables the model to focus more on hard-to-classify samples, which significantly improves its sensitivity to challenging cases and enhances the overall performance of the model. The formula for Focal Loss is as follows **Equation 5**.


(5) $$L_{\text{Focal}}=-\alpha_{t}{\left(1-p_{t}\right)}^{\gamma }\log(p_{t})$$

where:

$\alpha_{t}$ is the weight coefficient for positive and negative samples; $p_{t}$ is the model’s probability of predicting the correct category; and γ is the focal parameter that regulates the loss weights for easily categorized samples;

By incorporating CIOU and Focal Loss, YOLOv8 achieves simultaneous optimization in both regression and classification:

1). Regression optimization: CIOU enhances bounding box localization by introducing centroid distance and aspect ratio loss. This is particularly effective for detecting complex-shaped targets, improving the precision of bounding box regression.

2). Classification optimization: Focal Loss adjusts the loss weights of easy-to-classify samples, increasing the model’s focus on small targets and minority categories. This significantly boosts overall detection accuracy, particularly in imbalanced datasets.

This synergy between CIOU and Focal Loss demonstrates superior performance in experiments, effectively addressing the challenges of small target detection, overlapping frames, and category imbalance. In the tea leaf recognition task, where small buds and leaves are common and class distributions are imbalanced, this combination enhances YOLOv8’s robustness under varying planting environments, picking methods, and shooting conditions, ultimately achieving higher detection accuracy.

## 4 Experimentation and analysis

### 4.1 Comparison of model computation and robustness

The platform configuration for this model training experiment is: Windows 11, CPU: R9-7845HX running at silent frequency, GPU: RTX4060Laptop, RAM: 16G, hard disk: Samsung 1TB, Python-3.12.4 Pytorch-2.3.1+Cuda version 12.1.

**Figure 10:**
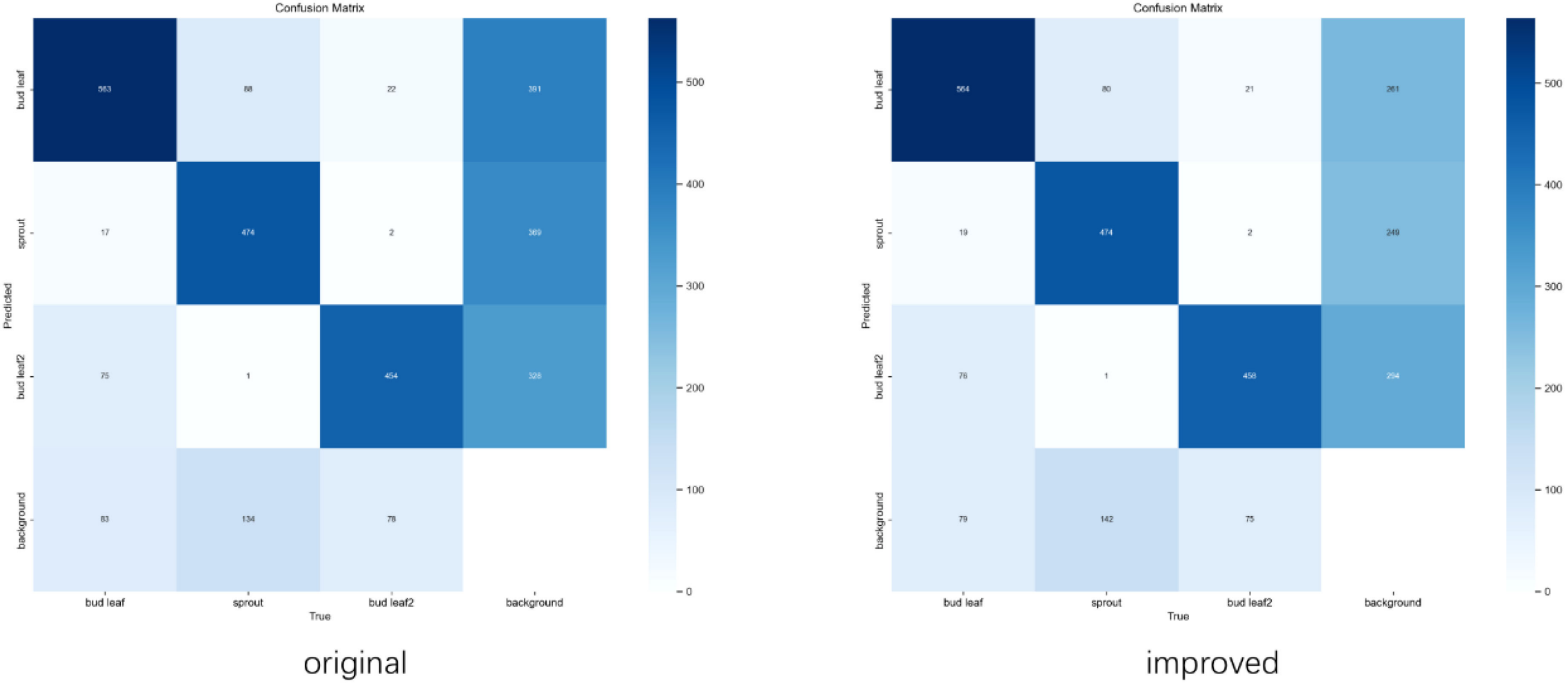
Confusion matrix.

From the comparison of the confusion matrices in **Figure 10**, the improved model significantly reduces the misidentification rate for the “background” category. Specifically, the number of background instances misclassified as “bud leaf” decreased from 391 to 261, while misclassifications into the “bud leaf2” and “sprout” categories dropped from 328 and 369 to 294 and 249, respectively. Additionally, correct classifications in the “bud leaf2” category increased from 454 to 458.

Overall, the improved model achieved a 26% reduction in the total false recognition rate, with notable improvements in distinguishing background noise from key categories. This reduction in background noise interference enhanced the model’s ability to recognize detailed features, leading to better overall performance and more accurate classifications in complex scenarios.

### 4.2 Ablation experiment

**Table 2** summarizes the comparison between the base model and the improved model in terms of the number of parameters and recognition accuracy. Despite the introduction of new loss functions, CBAM, and BiFPN modules, the model size only increases by 5%. However, the inference speed shows significant improvement, particularly in multi-target dense scenarios. The accuracy of the improved model reaches 80.5%, while the computational load is reduced by up to 19.3%. This enhancement in speed is attributed to the efficient fusion of multi-scale features and the attention mechanism, which filters redundant information and reduces computational costs.

**Table 2:** Table of ablation experiments.

Algorithm	CIOU	FOCAL	CBAM	BIFPN	P(%)	R(%)	mAp50(%)	GFLOPs	Model Size
1	×	×	×	×	70.5%	73.3%	75.7%	8.1	6.1
2	×	×	✓	×	72.7%	74.5%	76.9%	8.2	6.1
3	×	×	×	✓	73.5%	75.1%	77.2%	8.3	6.14
4	×	✓	×	×	75.6%	70.0%	75.9%	8.1	6.10
4	✓	×	×	×	76.2%	71.9%	76.6%	8.1	6.11
5	✓	✓	×	×	75.3%	74.4%	78.9%	8.1	6.12
6	✓	✓	✓	×	72.6%	75%	79.3%	8.3	6.11
7	✓	✓	✓	✓	74.4%	75.4%	80.5%	6.7	6.39

The results also highlight the critical role of CBAM and BiFPN in improving the model’s feature representation and fine-grained target detection accuracy. Removing any of these modules leads to a noticeable performance degradation, particularly when handling overlapping targets and complex backgrounds, where the accuracy drops more significantly. Specifically, CBAM enhances the attention mechanism, allowing the model to focus on important features, while BiFPN improves the detection of multi-scale targets through efficient feature fusion. These improvements contribute to a more robust and efficient detection system.

Overall, the results from the ablation experiments further validate the positive impact of the proposed improvements on model performance. The detailed experimental data provided in the paper demonstrate the effectiveness of CBAM and BiFPN in optimizing both accuracy and computational efficiency, reinforcing the credibility and practicality of our approach.

### 4.3 Comparison of tea recognition accuracy

To evaluate the performance improvement of the modified YOLOv8 model, the target detection accuracy before and after modification are compared. **Figure 10** illustrates the difference in detection effectiveness between the two models, emphasizing the enhanced performance of the modified model, especially in challenging scenarios such as dense target areas and small objects.

**Figure 11:**
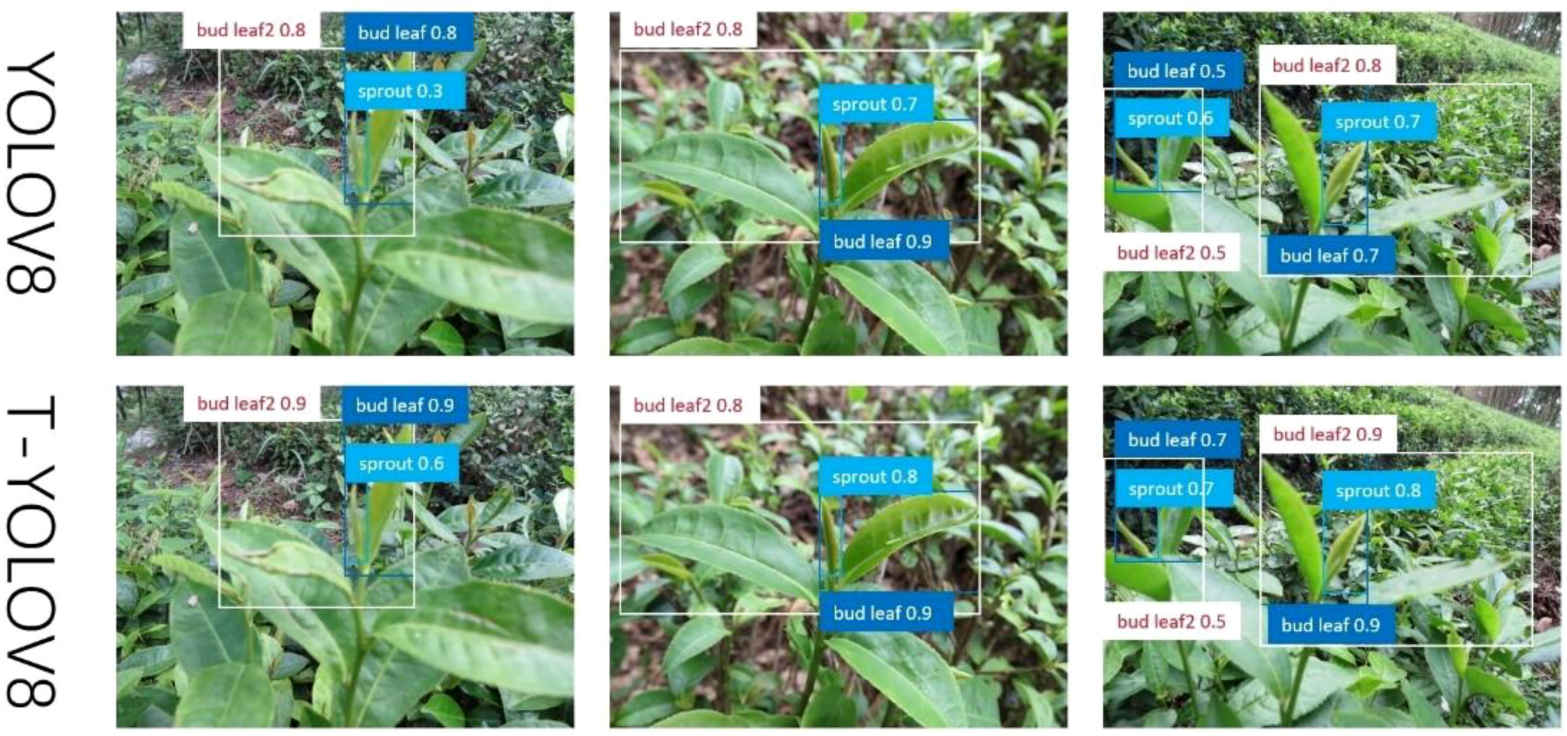
Accuracy comparison chart.

**Figure 11** compares the performance of the pre- and post-improvement models, revealing significant accuracy gains with the modified model. For instance, in the first group of images, the accuracy of the “sprout” class increases from 0.3 to 0.6, and “bud leaf” accuracy rises from 0.8 to 0.9. Similar improvements are observed in the other two groups, further demonstrating the model’s enhanced precision.

**Table 3** presents a comparison of several target detection models based on performance metrics including precision (P), recall (R), and computational complexity (GFLOPs). The modified T-YOLOv8n model is compared with YOLOv8n (Qiu et al., 2022; Liu et al., 2022; Jiang et al., 2024), YOLOv9-t (Li J. et al., 2024; Huang et al., 2024; Ye et al., 2024), and YOLOv5s (Ju et al., 2024; Zeng et al., 2023; Yar et al., 2023), which are representative models commonly used in target detection tasks. The results provide a clear visualization of the performance differences between T-YOLOv8n and other leading models.

**Table 3:** Comparison table of the performance of different models.

Model	P(%)	R(%)	mAP50(%)	GFLOPs	Model Size
T-Yolov8n	74.4%	75.4%	80.4%	6.7	6.39
Yolov8n	70.5%	73.3%	75.7%	8.1	6.1
Yolov9-t	75.5%	73%	77.5%	10.7	5.98
Yolov5s	72.5%	73.3%	71.6%	15.8	14.1

**Table 3** compares several target detection models, including the improved T-YOLOv8n, YOLOv9-t, and YOLOv5s, in terms of performance and resource efficiency. The T-YOLOv8n model significantly outperforms the base YOLOv8n, with an mAP@50 of 80.4%, a 4.7% increase compared to the original 75.7%. Precision improves from 70.5% to 74.4%, and recall increases from 73.3% to 75.4%, demonstrating a substantial boost in detection performance. In contrast, YOLOv9-t achieves a slightly higher precision of 75.5%, but its mAP@50 of 77.5% remains lower than that of T-YOLOv8n, and its computational cost (GFLOPs) is higher at 10.7, compared to T-YOLOv8n’s 6.7, making the latter more efficient in terms of computational resources.

Meanwhile, we consider the performance comparison with two traditional target detection models to further validate the effectiveness of the proposed method. the experiments revealed that both Faster R-CNN and SSD performed worse than the YOLOv8 series models on the dataset used in this study. This is mainly because our dataset contains overlapping annotations of multiple categories on the same tea leaves, and both Faster R-CNN and SSD exhibit significant limitations in handling such scenarios. Specifically, Faster R-CNN, as a two-stage detection model, relies on a proposal generation process for object localization, which, when faced with significant overlap between objects, often leads to category confusion or missed detections. SSD, on the other hand, has a relatively simple feature fusion mechanism, making it difficult to accurately localize and classify fine-grained objects in complex backgrounds. As a result, the performance of these two traditional models on this dataset did not reach the desired level.

The experimental results show that Faster R-CNN achieved an mAP of 72.3% and SSD an mAP of 70.8%, both of which are lower than the 75.7% mAP of YOLOv8n. In contrast, the improved T-YOLOv8n, with the introduction of CBAM BiFP and optimization of the loss function, further increased the mAP to 80.4%, effectively demonstrating the validity of the proposed method.

As shown in **Table 4**, compared to traditional object detection models, T-YOLOv8n achieves a significant improvement in accuracy. This result indicates that T-YOLOv8n is better suited to handle the multi-object overlapping annotation scenarios in the dataset used in this study and demonstrates higher practicality in complex detection tasks.

**Table 4:** Comparison table of the performance of different stage models.

Model	Type	mAP(%)
Faster-rcnn-pytorch	Two-stage Model	72.3
SSD.Pytoch-master	One-stage Model	70.8
YOLOv8n	One-stage Model	75.7
T-YOLOv8n	One-stage Model	80.5

**Figure 12:**
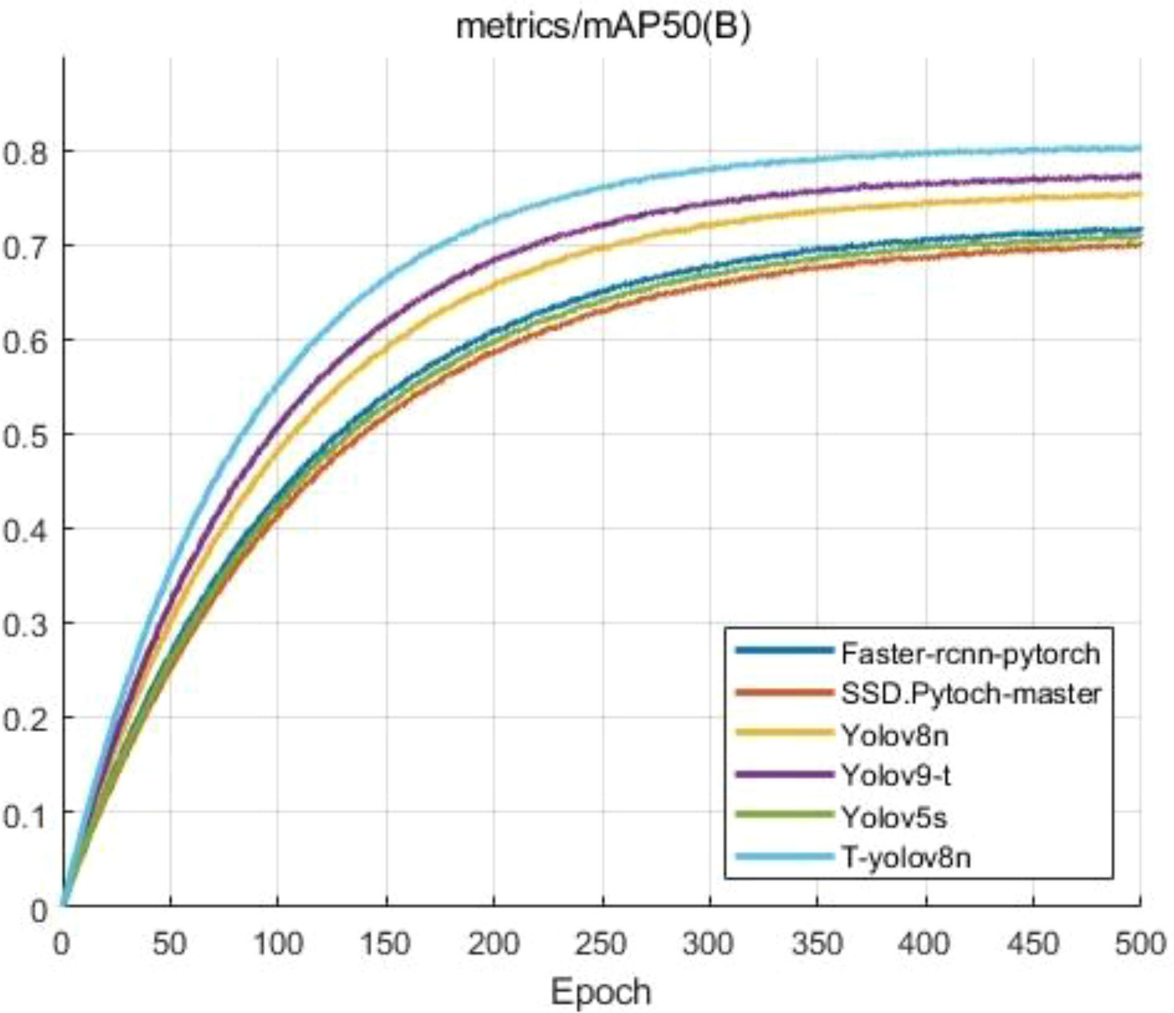
Comparison of different models on mAP@50 performance.

The data in the above table shows that, YOLOv5s, with a model size of 14.1 MB and GFLOPs of 15.8, demonstrates the weakest performance with an mAP@50 of only 71.6%. Its larger model size and higher computational complexity result in significantly worse performance compared to YOLOv8. This shows that YOLOv5s is less efficient and unsuitable for deployment in resource-constrained environments. In contrast, the T-YOLOv8n model strikes an optimal balance between performance and efficiency, achieving significant improvements in mAP@50 and accuracy, while reducing both model size and computational complexity. For traditional target detection models, the Faster-RCNN model, a two-stage detection model, achieves an mAP of 72.3%, while the SSD model, another one-stage model, delivers a slightly lower mAP of 70.8%. YOLOv8n, a more advanced one-stage model, performs better with an mAP of 75.7%. In contrast, the T-YOLOv8n model achieves the highest performance, with an mAP of 80.5%, demonstrating its superior accuracy and efficiency compared to traditional models. The performance differences between these models are clearly shown in **Figure 12**, which visually compares mAP@50.

As shown in **Figure 12**, the mAP@50 of the different models changes as the training epochs increase. The improved T-YOLOv8n model (light blue curve) demonstrates the best performance growth throughout the training process. It converges faster in the early stages, stabilizing around 150 epochs and eventually achieving an mAP@50 close to 0.8. In contrast, YOLOv8n (yellow curve) and YOLOv9-t (purple curve), although starting with similar performances, both exhibit lower mAP@50 values than T-YOLOv8n in the later stages, remaining around 0.75 and 0.77, respectively. YOLOv5s (green curve) performs the worst in the YOLO’s family, with its mAP@50 stabilizing around 0.7 and showing slower convergence. The SSD model (orange curve) follows closely beneath YOLOv5s, with slightly lower mAP@50 values throughout the training process. Meanwhile, the RCNN model (blue curve), although initially performing better than SSD and YOLOv5s, shows a relatively slower improvement, eventually stabilizing just above YOLOv5s at around 0.73.The figure clearly highlights T-YOLOv8n’s superior performance in training, not only reaching a higher mAP@50 earlier but also maintaining better accuracy at the end of training. This demonstrates the improved model’s robustness, faster convergence, and greater efficiency.

## 5 Conclusions

In this paper, a tea category detection method based on the improved YOLOv8 model is proposed, which significantly improves the model’s detection precision and robustness by integrating modules such as CBAM, BiFPN, CIOU, and Focal Loss. Experimental results show that the improved YOLOv8 model excels in tea small target detection and handling overlapping targets, achieving precision (P), recall (R), and mAP@50 scores of 74.4%, 75.4%, and 80.4%, respectively. These results represent a notable performance improvement compared to the original model. Moreover, the combination of online data enhancement and Mosaic enhancement strategies further boosts the model’s generalization ability, especially under complex background and lighting conditions.

When compared to other mainstream detection algorithms (e.g., YOLOv5 and YOLOv9), the improved algorithm demonstrates clear advantages in detection accuracy, particularly in handling fine and overlapping tea leaves. At the same time, it maintains low computational complexity, making it well-suited for practical applications such as tea garden detection. Despite the slight increase in parameters and computation, the model remains lightweight, ensuring smooth deployment on small devices. In practical field applications, although the model’s performance is satisfactory, there are still some challenges. For example, complex natural environments (such as strong sunlight, shadows, wind-blown leaves, etc.) may affect detection accuracy. Additionally, the deployment efficiency of the model on resource-constrained devices still requires further optimization to ensure real-time detection on embedded devices or mobile platforms.

In the future, we believe that improvements can be made in the following three areas. First, expanding the dataset’s diversity to cover tea garden images from different seasons and climate conditions, which will enhance the model’s robustness in complex environments. Second, while the current model primarily uses static images, we plan to explore real-time data processing on larger tea plantations in future work. This will include testing the model on more powerful hardware configurations to improve scalability and performance under dynamic conditions such as wind and changing light. Third, although we have simulated dynamic changes through data augmentation (e.g., brightness, contrast, saturation adjustments, and random geometric transformations), we plan to conduct further research on real-time video stream analysis to optimize the model’s performance in dynamic environments.

Additionally, the deployment of the model on resource-constrained devices will require further research into optimizing hardware configurations. Though the current study does not focus on this aspect, future research will explore efficient hardware deployment to enhance the real-time application of the model. Last, integrating multi-task collaboration, such as disease detection and drone inspections, to further improve the efficiency and accuracy of intelligent tea garden management. In the meantime, we plan to expand the dataset in future work to include images of tea gardens from different regions and under different climatic conditions, thereby improving the adaptability and stability of the model in a wider range of environments.

Through these efforts, the findings of this study are expected to be widely applied in real-world production, providing important technical support for the intelligent and precise management of tea gardens.

## Data Availability

The data analyzed in this study is subject to the following licenses/restrictions: Access to the dataset is restricted to ensure compliance with data protection regulations. Interested researchers may contact the corresponding author for further information and potential access. Requests to access these datasets should be directed to tangli@mail.xhu.edu.cn.
